# Antimicrobial Formulation of a Bacterial Nanocellulose/Propolis-Containing Photosensitizer for Biomedical Applications

**DOI:** 10.3390/polym15040987

**Published:** 2023-02-16

**Authors:** Isabella Salgado Gonçalves, Lais Roncalho Lima, Andresa Aparecida Berretta, Nathaly Alcazar Amorim, Sebastião Pratavieira, Thaila Quatrini Corrêa, Flávia Aparecida Resende Nogueira, Hernane Silva Barud

**Affiliations:** 1 Laboratório de Biopolímeros e Biomateriais—BioPolMat, University of Araraquara, Araraquara 14801-320, SP, Brazil; 2 Exact Sciences and Technology Center, Federal University of São Carlos, São Carlos 13565-905, SP, Brazil; 3 Physics Institute of São Carlos, University of São Paulo, São Carlos 05508-060, SP, Brazil; 4 Chemistry Department, Federal University of São Carlos, São Carlos 13565-905, SP, Brazil; 5 Chemistry Department, Federal University of Maranhão, São Luís 65080-805, MA, Brazil; 6 Research, Development and Innovation Department, Apis Flora Indl. Coml. Ribeirão, Preto 14020-670, SP, Brazil

**Keywords:** bacterial nanocellulose, propolis, hydrogels, photodynamic inactivation, wound healing, antimicrobial

## Abstract

With the aim of contributing to the development of more efficient materials for wound care, new topical formulations based on bacterial nanocellulose (BNC) hydrogels containing propolis were produced. Characterizations confirmed the incorporation of propolis into the BNC matrix, maintaining its structure and properties. Rheological analysis confirmed that the hydrogels showed thixotropic behavior appropriate for topical application. Chromatographic profiles showed sustained release of propolis biomarkers for at least 20 h. The formulations did not present mutagenicity. For application in photodynamic inactivation (PDI), BNC/propolis hydrogels were prepared with the photosensitizers methylene blue (MB). Spectroscopy and confocal fluorescence microscopy confirmed the interaction of MB and propolis in BNC hydrogels, as well as the formation of a new composite material. In the antibacterial assays, formulations containing MB and propolis significantly reduced *Staphylococcus aureus* growth. In the presence of light, BNC/MB hydrogels completely inhibited the microorganism. Therefore, the results suggest potential materials for the prevention or treatment of *Staphylococcus aureus* infections in wounds.

## 1. Introduction

Wound healing is a complex and dynamic process influenced by local and systemic factors that involve reactions and interactions between cells, biomolecules, and mediators occurring in proper sequence and time [1]. The normal healing process is traditionally divided into the integrated and overlapped phases of inflammation, proliferation, and remodeling [2]. However, normal physiologic responses can be affected by many factors, which may impair progress and lead to delayed acute wounds and chronic wounds [3].

Infection is one of the main causes of delay or impediment in wound repair because it generates an intense immune response and tissue damage as a consequence of excessive microbial proliferation at the injury site. In addition, the bacterial biofilm present in most chronic wounds hinders the penetration of antimicrobials and contributes to antibiotic tolerance and resistance, which further complicate treatment [4,5]. Chronic wounds represent a significant clinical challenge for patients and healthcare professionals due to the long periods of treatment, persistent infections, and high recurrence rates, making them an important issue for the healthcare system [6,7].

Traditional treatments for wound infections are based on systemic or topical administration of antimicrobial and antiseptic agents, which are, in general, associated with dressings or coverings in the form of hydrogels, hydrocolloids, polymeric films, gauze, foams, and alginates [4,8,9]. However the high failure rate associated with increased antimicrobial resistance brings about the need to develop more effective treatments with advanced technologies [10].

In this sense, bacterial nanocellulose (BNC), a notable biopolymer that is mainly secreted by bacteria of the *Komagataeibacter genus*, is already used as dressing in the treatment of wounds and can act as a temporary skin substitute [11,12,13]. BNC is produced in the form of highly hydrated membranes with three-dimensional nano- and microfiber lattice structures [14,15]; it presents advantageous properties for wound care, such as maintenance of moisture in the wound bed, absorption of excess exudate, fluid exchange control, pain relief, and protection against microorganisms, due to its nanometric network, which acts as a barrier [11,12,16]. In order to add antimicrobial activity to BNC membranes, the incorporation of antimicrobial agents, e.g., silver nanoparticles [17,18], antibiotics [19], and natural compounds such as propolis, has been studied [20]. BNC combines the diverse properties of hydrogels with biocompatibility and resemblance to living tissue, allowing for broad opportunities for biomedical applications, particularly in drug delivery and release systems, wound dressings, and tissue engineering [21].

Propolis, or bee glue, is a resinous product produced by *Apis mellifera* bees from several plant materials, including sprouts and exudates. This product, which is originally used to fix and protect honeycombs, has also been recognized for centuries in terms of its biological and pharmacological properties [22,23]. Propolis extracts have been widely used in medicine due to their antiseptic, antimicrobial [24,25], anti-inflammatory, immunomodulatory [26,27], antioxidant [28], analgesic [27], antitumoral [29], and anticancer [30] properties, as well as wound-healing activities [24,31], among others. Previous studies have demonstrated the efficacy of free-standing BNC films containing standardized propolis extract (EPP-AF^®^), ethanol extract [20], and EPP-AF^®^ microemulsions [32] against *Staphylococcus aureus* and *S. epidermidis* bacteria, further confirming the healing effect of these composites on wounds.

In the context of wound treatment, many groups have used light as an agent to promote wound healing through effects of photobiomodulation [33,34,35], which is a potent, safe, and noninvasive technique [36]. When associated with a photosensitive agent (PS) and molecular oxygen (O_2_), light at a specific wavelength causes photochemical and photophysical reactions originating photodynamic reactions [37]. The mechanism of photodynamic reactions is based on the absorption of light energy by PS electrons and their interaction with surrounding molecules, originating reactive oxygen species (ROS). In type I reactions, an excited PS transfers electrons or hydrogen atoms, generating oxidative radicals, such as superoxide anion (O_2_^-^), hydrogen peroxide (H_2_O_2_), and hydroxyls (OH^−^). Type II reactions involve energy transfer to O_2_, producing singlet oxygen (^1^O_2_) [38]. These reactive species have high oxidizing power and can damage proteins, lipids, nucleic acids, and other molecules present in cells, resulting in cellular death by necrosis or apoptosis [39].

The first studies and application of photodynamic reactions were related to the treatment of several types of tumors, including skin, gynecological, head and neck, esophageal, and gastric cancers [40,41]. Subsequently, photodynamic reactions were applied against microorganisms and for infection control, with dominance of photodynamic inactivation (PDI), antimicrobial photodynamic therapy (aPDT), or photodynamic antimicrobial chemotherapy (PACT). These reactions can effectively treat localized infections without causing microbial resistance [41,42,43]. The success of PDI has been demonstrated in treating throat infections [44], oral candidiasis [45], onychomycosis [46], acne, and other skin injuries caused by microorganisms [47]. It has exhibited benefits in wound care by reducing infection that delays healing [48,49].

Methylene blue (MB) is an organic dye derived from phenothiazine that has fluorescent and photosensitizing properties. Therefore, it is widely used in PDI as a photosensitizer. MB can eliminate malignant cells and inactivate viruses, fungi, and bacteria through photodynamic reactions [50,51].

In this work, we report the development and characterization of new BNC hydrogel-based formulations containing propolis and MB. The advantage, compared to conventional dressings (free-standing films or membranes), is that the hydrogel allows for better spreading and dispersion over the infected areas, in addition to enabling applications in more complex wounds that have tunnels, adhering the material throughout the targeted area (due to adequate rheological properties). In view of existing dressings reported in the literature based on BNC and propolis, this study presents the progress of several treatments, mainly with respect to the applicability of hydrogels based on BNC and in association with photodynamic therapy. From a hydrogel-based material, new properties can be obtained, such as high exudate capacity and non-adherence (easily removed from the wound), in addition to being easily developed and handled. Specifically, the material proposed in this work allows for the use of light to enhance treatment against microorganisms around wounds. All BNC hydrogels were fully characterized by scanning electron microscopy (SEM), Fourier transform infrared spectroscopy (FTIR), thermogravimetric analysis (TGA), and rheology. We also elaborated a drug delivery profile and evaluated in vitro antimicrobial activity, including confocal photodynamic inactivation against *S. aureus*.

## 2. Materials and Methods

### 2.1. Bacterial Nanocellulose Pulp

Wet BNC was obtained from static cultivation of the bacterium *Komagataeibacter rhaeticus* in Hestrin and Schramm (HS) liquid medium composed of 50 g L^−1^ glucose, 4 g L^−1^ yeast extract, 0.73 g L^−1^ magnesium sulfate (MgSO_4_), 20 mL ethanol, and 980 mL distilled water. After 5 days of incubation at 28 °C, the membranes were treated in a 0.1 M sodium hydroxide (NaOH) solution to remove residues and washed in water until reaching neutral pH. The hydrated BNC membranes were ground in a high-speed dispersing element (Ultraturrax) under aqueous conditions. The suspension of BNC microparticles was sieved, resulting in a BNC pulp.

### 2.2. Hydrogel Production

Hydrogels were prepared by mechanical stirring of BNC pulp with 1% of the gelling agent Natrosol^®^ (hydroxyethyl cellulose), 0.18% and 0.02% of the preservatives Nipagin^®^ (methylparaben) and Nipazol^®^ (propylparaben), respectively, and 5% of the humectant propylene glycol, all measured in *w*/*w* relative to the total mass (100%) of the hydrogel. Standard BNC hydrogel containing 1% dry mass of BNC was produced, named BNCH. From BNCH, formulations were prepared by adding 1.2, 2.4, and 3.6% (*w*/*w*) propolis extract (EPP-AF^®^) provided by Apis Flora Company (Ribeirão Preto, São Paulo, Brazil). The samples were named BNCH/P1, BNCH/P2, and BNCH/P3, respectively. Propolis concentrations were based on the study of Berretta et al. (2012) [24]. Natrosol^®^ hydrogel was also produced as a control group and named BH.

For photodynamic inactivation, BNCH/P1 hydrogels were reproduced with the addition of MB at concentrations of 0.01 and 0.1% (*w*/*w*), originating samples BNCH/P1/MB1 and BNCH/P1/MB2, respectively. Formulations containing only BNC hydrogel and MB (BNCH/MB1 and BNCH/MB2) were also produced, keeping the same reagent concentrations.

### 2.3. Characterization of Bacterial Nanocellulose Hydrogels Containing Propolis

A JEOL field-emission scanning electron microscope (JMF-6700F–Field Emission SEM/Analytical Field Emission SEM) was used to evaluate the morphology and microstructure of the BNC hydrogels and observe the influence of propolis on the formation of composites. For the analysis, freeze-dried samples were coated with a carbon layer and placed on copper supports.

FTIR spectra were obtained to confirm the molecular interaction between propolis and the BNC matrix using Cary 630 FTIR equipment (Agilent Technologies, Santa Clara, CA, USA) in the range of 650 to 4000 cm^−1^ with 64 scans and a 4 cm^−1^ resolution. Measurements were performed with the freeze-dried formulations.

Thermogravimetric analysis was performed to evaluate the thermal behavior of BNC/propolis hydrogels using an SDT Q600 instrument (TA Instruments, New Castle, DE, USA). The freeze-dried samples were heated at a constant rate of 10 °C min^−1^ from 30 °C to 600 °C under a nitrogen flow of 100 mL min^−1^.

Flow curves were determined by rheological assays conducted at room temperature (25 °C) using a TA Instruments rheometer (AR1500) equipped with a 2° cone geometry. The shear rate ranged from 0 to 280 Pa s^−1^ for the upward flow for 120 s and from 280 to 0 Pa s^−1^ for the downward flow for 120 s. The results were analyzed by TRIOS software v 5.1.1.

Chemical characterization of propolis extract was carried out by high-performance liquid chromatography (HPLC) using a Shimadzu (Kyoto, Japan) liquid chromatograph equipped with a CBM-20A controller, an LC-20AT quaternary pump, an SPD-M 20A diode-array detector, and Lab Solution Software version 5.92. A Shimadzu Shim-Pack column (4.6 mm × 250 mm, particle diameter of 5 µm, and pore diameter of 100 Å) was used. The mobile phase consisted of methanol (B) and a solution of water–formic acid (0.1% *v*/*v*), pH 2.7 (A). A linear gradient of 20–95% of B was used for 77 min at a flow rate of 0.8 mL min^−1^, with an injection volume of 10 μL. The column oven was set to 40 °C, and detection was set at 275 nm. The compounds were identified by comparison with chromatographic standards *p*-coumaric acid obtained from Sigma Aldrich (Saint Louis, MO, USA); artepillin C from Wako Chemical Industries Co. (Osaka, Japan); and baccharin isolated, purified, and supplied by Professor Jairo Kenupp Bastos from the University of São Paulo. For these analyses, the formulations were diluted in methanol (HPLC grade) and 0,1% formic acid (1.5:1). The samples were filtered through a 0.45 µL filter before injection.

Release studies were performed in vitro using a Franz diffusion cell at 37 °C. The receptor compartment was filled with phosphate buffer (pH 7.5), 0,9% sodium chloride (NaCl), and 12% hydrogenated ethoxylated castor oil (Cremophor RH40 OE). Then, 500 mg of the hydrogels was deposited in the donating compartment, and 500 µL of receptor solution samples was collected from the Franz cell at predetermined intervals, followed by replacement with the same volume of pure receptor solution. Samples were analyzed using the HPLC methodology to quantify *p*-coumaric acid and artepillin C, both of which are propolis biomarkers. Mathematical dilution adjustments were considered to correctly determine the content of the markers in the samples. The experiments were carried out in duplicate, and the propolis release profiles were determined. To evaluate the release kinetics of biomarkers, zero-order, first-order, and Higuchi mathematical models were applied.

Mutagenic potential analysis was accomplished according to the preincubation methodology developed by Maron and Ames (1983) [52] using TA98, TA100, TA102, and TA97a strains of *Salmonella typhimurium* in experiments with (+S9) and without (−S9) metabolic activation. The S9 microsomal fraction, a freeze-dried Sprague–Dawley rat liver homogenate, was purchased from Moltox Molecular Toxicology Inc. (Boone, NC, USA). Hydrogels were solubilized in dimethyl sulfoxide (DMSO), reaching the non-toxic concentration of 0.01 g mL^−1^ [53]. After defrosting, the bacterial stock culture was sown in Oxoid Nutrient Broth nº 2 and incubated for 18 h (overnight) at 37 °C, obtaining a density of 1–2 × 10^9^ bacteria mL^−1^. Then, 0.1 mL of this bacterial culture, 0.5 mL of 0.2 M phosphate buffer, or 0.5 mL of 4% S9 mixture was added to the samples, followed by incubation. Subsequently, 2 mL of top agar supplemented with histidine and biotin was added to the tubes and plated on minimal agar. After 48 h of incubation at 37 °C, the number of histidine-revertant colonies per plate (His^+^) was counted manually. All tests were performed in triplicate. The standard mutagens used to confirm the reversion properties and specificity of each strain in experiments without the S9 mixture were 4-nitro-o-phenylenediamine (10.0 µg/plate) for TA98 and TA97a, sodium azide (1.25 µg/plate) for TA100, and mitomycin C (0.5 µg/plate) for TA102. In the experiments with metabolic activation, 2-aminoanthracene (1.25 µg/plate) was used for TA98, TA97a, and TA100, and 2-aminofluorene (10.0 µg/plate) was used for TA102. DMSO was used as negative control (100 µL/plate). Data from the Ames test were expressed as mean ± standard deviation (SD), and the statistical significance was determined by one-way analysis of variance (ANOVA) complemented by Dunnett’s test (post hoc test) to compare the results with those of the control group (DMSO). The mutagenic index (MI) was also calculated as the average number of revertants per test plate divided by the average number of revertants per solvent (negative control plate). A sample was considered mutagenic if the ANOVA variation was significant, with *p* < 0.05 and an average increase in revertants in the sample of a minimum of two folds of that found in the negative control (MI > 2) [54].

### 2.4. Characterization of Bacterial Nanocellulose Hydrogels Containing Propolis/Methylene Blue

Ultraviolet and visible (UV-Vis) absorption spectroscopy was performed to characterize the absorption profile of the BNC hydrogels containing the photosensitizer MB and the influence of propolis on its behavior. The scanning range was 350 to 800 nm. The hydrogels, in their natural form, were spread over glass slides (1 mm thick) and analyzed in a Varian Cary^®^ 50 Bio spectrophotometer.

For fluorescence analysis, a portable prototype fluorescence spectroscopy system was used, consisting of a spectrophotometer (USB2000, Ocean Optics–Palo Alto, CA, USA, a “Y” type fiber Ocean Optics), a light source, filters, and a computer. The excitation was provided by a 405 nm diode laser, and the fluorescence emission was acquired by the spectrophotometer, which was connected to a computer using OOIBase software (Ocean Optics, Orlando, FL, USA) [55].

To evaluate the morphology and spatial fluorescence characteristics of BNC/MB hydrogels and their behavior in the presence of propolis, a Zeiss–LSM780 inverted confocal fluorescence microscope was used. The samples were excited by a diode laser emitting at 405 nm with a power of 40 μW cm^−2^ and operating in continuous (*cw*) and pulsed mode. The pixel dwell was fixed at 30 μs, and fluorescence spectra were acquired in the range of 400–700 nm (resolution of 8 nm) by high-sensibility GaAsP photomultipliers. For the assay, the samples were previously freeze-dried and analyzed on glass slides [56].

The antimicrobial photodynamic activity of hydrogels was investigated against *Staphylococcus aureus* (ATCC 25923) using microdilution and the agar diffusion methods. The experimental groups were BNCH/P1, BNCH/P1/MB1, BNCH/P1/MB2, BNCH/MB1, and BNCH/MB2, and the control groups were BH and BNCH. The assays were performed with and without light irradiation at 660 nm.

*Staphylococcus aureus* was cultivated in tryptic soy broth (TBS). To this end, 7–8 bacterial colonies were inoculated in 10 mL of TSB and incubated at 37 °C overnight. Then, 500 µL of inoculum was transferred to 9.5 mL of fresh TBS, and this sample was incubated at 37 °C for 4 h to reach the mid-log phase. The turbidity of the bacterial suspension was adjusted in a spectrophotometer at 600 nm to obtain 10^8^ colony-forming units per mL (CFU/mL).

For the microdilution method, the bacterial suspension was centrifugated at 3000 rpm for 15 min and resuspended in phosphate-buffered saline (PBS). Then, 0.15 g aliquots of each hydrogel were transferred into 24-well plates, followed by 400 μL aliquots of the bacterial suspension in PBS. After homogenization, the samples were left in the dark for 20 min at room temperature, then irradiated by red light at 660 nm with a light dose of 50 J cm^−2^. Later, 100 μL aliquots of each sample were diluted in 900 μL of PBS, and five 10-fold serial dilutions were carried out for each sample. Finally, a volume of 15 μL from each dilution was streaked onto brain–heart infusion (BHI) agar plates according to the spread plate method. Plates were incubated at 37 °C for 24 h for the bacterial count. The agar diffusion method was also used to verify the antimicrobial photodynamic activity of hydrogels.

Based on the CLSI disk diffusion method, the freeze-dried hydrogels were cut into 1 cm^2^ films. Then, 100 μL aliquots of the bacterial suspension were streaked onto BHI agar plates according to the spread plate method. After drying, the pieces of hydrogel films were placed in the center of each Petri dish quadrant. The plates from the experimental groups were irradiated at 660 nm with a light dose of 50 J cm^−2^, and, in the end, all cultures, including the control groups that were not illuminated, were incubated at 37 °C for 24 h. The inhibition zone halo was measured with a ruler, and the values were noted in millimeters. All experimental antimicrobial studies were performed in triplicate.

Statistical analysis of the photodynamic inactivation results was performed using Origin software, and the data were evaluated by one-way analysis of variance (ANOVA). The differences between all experimental groups were evaluated by ANOVA, followed by Tukey’s post hoc test, with the significance level set at *p* < 0.05.

## 3. Results and Discussion

### 3.1. Characterization of Bacterial Nanocellulose Hydrogels Containing Propolis

#### 3.1.1. Scanning Electron Microscopy (SEM)

**Figure 1:**
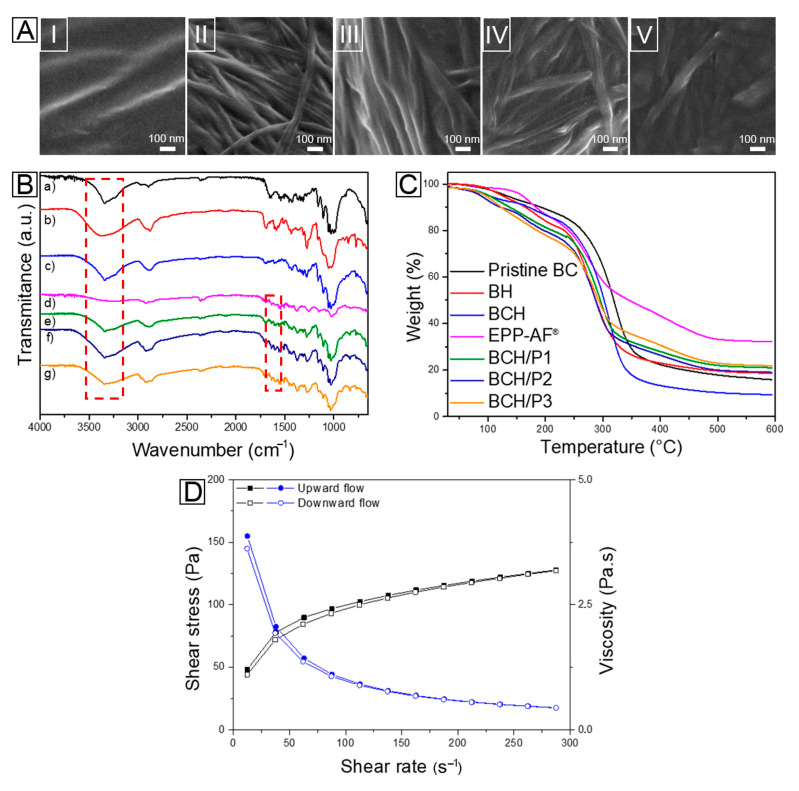
(**A**) SEM images of (**I**) BH, (**II**) BNCH, (**III**) BNCH/P1, (**IV**) BNCH/P2, and (**V**) BNCH/P3. (**B**) FTIR spectra of (**a**) pristine BNC, (**b**) BH, (**c**) BNCH, (**d**) EPP-AF^®^, (**e**) BNCH/P1, (**f**) BNCH/P2, and (**g**) BNCH/P3. (**C**) TGA curves. (**D**) Flow curve of a representative BNC-based hydrogel.

Electron microscopic images of the formulations (Figure 1A) show a large quantity of micro- and nanofibers in samples containing BNC (from II to V), in contrast to the control group (I), which presents an evidently homogenous aspect. The hydrogels containing propolis maintained the native BNC structure of a three-dimensional network formed by its nanofibers. The fibrils remain in a random arrangement with a high degree of interlacing, which confirms the classical porous structure of BNC [57]. In general, the diameter of nanofibers ranges from 20 to 100 nm [58]. Figure 1(AV) shows a more compact fiber structure compared to the other formulations, suggesting the effective incorporation of propolis into the biopolymer matrix. A higher concentration of propolis in the hydrogels results in a uniform filing of the BNC nanometric pores.

#### 3.1.2. Fourier Transform Infrared Spectroscopy (FTIR)

According to the spectra (Figure 1B), all samples present with a band around 3300 cm^−1^, characteristic of OH stretching vibration, which was more intense in hydrogels containing BNC due to its greater water absorption. The bands at 1680 cm^−1^ and 1700 cm^−1^ correspond to the 2 C=O and C=C stretching of the flavonoids and aromatic rings from other phenolic compounds of propolis, respectively [59]. Both vibrations are present in the spectrum of pure EPP-AF^®^ and in the hydrogels that contain it, indicating the incorporation of propolis in the formulations, which corroborates the SEM results. Another typical band that confirms propolis in composite hydrogels occurs around 830 cm^−1^, referring to the angular deformation of aromatic rings [60].

In the region of 2850 cm^−1^, there is a discrete band characteristic of EPP-AF^®^, which is repeated in hydrogels and becomes more pronounced in the formulation with the highest concentration of propolis (BNCH/P3). The bands between 2850 and 2970 cm^−1^ in the pristine BNC, BH, and solid EPP-AF^®^ spectra were superimposed on the hydrogels, indicating a possible interaction between the components and composite formation.

#### 3.1.3. Thermogravimetric Analysis (TGA)

As shown in Figure 1C, BNC exhibits a typical thermal profile, which consists of two main events. In the first event, there is a small loss of mass (10%) in the temperature range of 30 to 150 °C, which is attributed to water loss. The second event occurs between 250 °C and 350 °C and results in a large mass loss (around 70–80%) related to the depolymerization of cellulose and the decomposition of glycosidic units, followed by the formation of carbonic residues [20,61]. BNC hydrogel (BNCH) presented a thermal profile similar to that of pristine BNC. However, the first degradation step occurs between 25 °C and 200 °C and comprises other mass loss events, probably due to the presence of pure hydrogel components (BH). Natrosol^®^ (hydroxyethyl cellulose) is an ester derived from cellulose, the thermal behavior of which is similar to that of BNC but with slightly lower thermal stability [62].

The EPP-AF^®^ curve shows associated events of propolis component decomposition, either successive and/or simultaneous, at room temperature with 5% loss. This event is related to the evaporation of volatile compounds present in the extract [63]. Much of the propolis degradation occurs between 200 °C and 500 °C, with mass loss of around 60% (at 275 °C), which is attributed to the carbon bond breakdown and degradation of organic compounds. BNC/propolis hydrogels presented intermediate thermal behavior between BNCH and EPP-AF^®^, with a slight decrease in thermal stability. According to Barud et al. (2013) [20], the degradation of formulations starts only at high temperatures, enabling application to the skin. The hydrogels start to lose mass continuously at a maximum rate around 290 °C, ending at 550 °C. These events are related to the condensation of OH groups, the breaking of carbon bonds, and the degradation of organic compounds from propolis, as well as the degradation of BNC [63,64].

It is noteworthy that the TGA curve of the hydrogel with the highest concentration of propolis (BNCH/P3) is similar to the profile of pure propolis, while the thermal behavior of the hydrogel with the lowest proportion of the extract (BNCH/P1) corresponds to the mass loss events of BH and BNCH. These data corroborate the SEM and FTIR results, confirming the new composite formulation.

#### 3.1.4. Rheological Analysis 

Rheological flow properties are related to the deformation of the material when it is subjected to shear stress, providing information about its behavior and stability, which is fundamental for quality control, material acceptance, and effectiveness [65,66]. All formulations showed non-Newtonian behavior, since there was no linear proportional ratio between shear stress and shear rate. Figure 1D shows the flow curve of a representative sample of a BNC-based hydrogel. In terms of rheological behavior, the flow curve shows the pseudoplastic behavior that results from the alignment of the disrupted three-dimensional network in the system in the direction of flow, providing the protective film formation characteristic that allows the skin surface to be covered, promoting improved protection [66].

The viscosity of hydrogels decreases with increasing shear rate. When this rate is removed, the hydrogels gradually recover their values through microrestructuring of formulation elements. Furthermore, an area of hysteresis is formed between the upward and downward flow curves, which characterizes thixotropic behavior [67]. This is an important characteristic for topical application formulations due to the ease of removing the contents of the package and allowing the product to be spread on the skin, preventing runoff [68,69]. Materials with thixotropic characteristics also tend to have increased shelf life, as they maintain constant viscosity during storage, avoiding the separation of constituents from the formulations [66].

**Figure 2:**
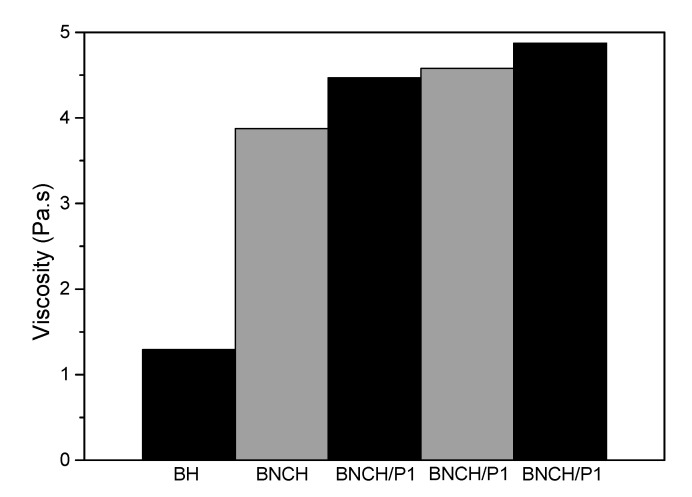
Viscosity of all BC-based hydrogel formulations. The viscosity is dependent on propolis content.

In general, in the presence of BNC, there is an increase in complex viscosity compared to pure BH for all hydrogels. Specifically, for formulations containing propolis, the behavior is very similar between samples with different concentrations (BNCH/P1, BNCH/P2, and BNCH/P3). There is a slight gradual increase in viscosity associated with an increase in propolis contents (Figure 2). Furthermore, there is an overlapping of curves with similar values for all samples, i.e., the rheological properties remain comparable with the same inclination values.

#### 3.1.5. Chemical Profile of EPP-AF^®^ Biomarkers and Release Study

**Figure 3:**
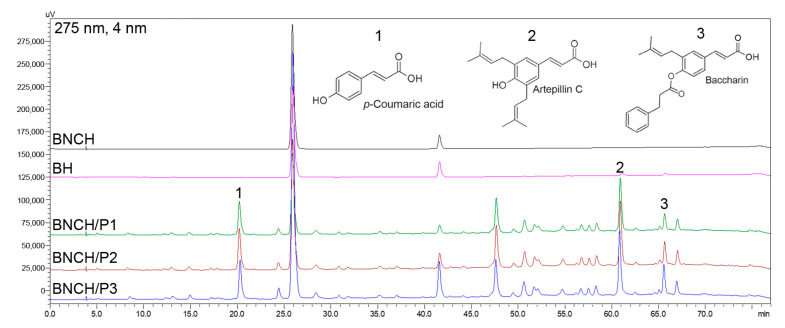
Chromatographic profile of hydrogels: (**1**) *p*-coumaric acid, (**2**) artepillin C, and (**3**) baccharin.

A chromatographic analysis of biomarkers from EPP-AF^®^ was performed to confirm the composition of propolis extract according to the methodology previously published by Berretta et al. (2012) [24]. The obtained chemical profile (Figure 3) identifies characteristic peaks of (1) *p*-coumaric acid, (2) artepillin C, and (3) baccharin, the amounts of which are listed in Table 1.

The results are comparable to the chemical profile of EPP-AF^®^ [24], confirming the proper incorporation of the extract into the formulations. As expected, the negative controls, BH and BNCH, do not present characteristic peaks of propolis.

**Figure 4:**
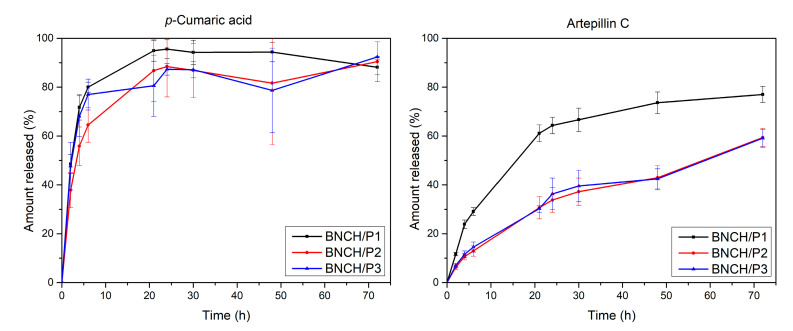
Release profiles of *p*-coumaric acid (**left**) and artepillin C (**right**) as percentage release over time.

The amounts of *p*-coumaric acid and artepillin C identified in the hydrogels are evidently proportional to the concentration of propolis extract used in each formulation (BNCH/P1–1.2% EPP-AF^®^, BNCH/P2–2.4% EPP-AF^®^, and BNCH/P3–3.6% EPP-AF^®^). However, the proportion was not maintained for baccharin, possibly due to its degradation during the protocol, as well as lower stability or greater difficulty in extracting the compound. The release study was carried out with propolis hydrogels to evaluate its biomarker release over time (Figure 4).

Although artepillin C is present in the propolis extract in its highest concentration according to the chromatographic profile, it was released in lower proportions compared to *p*-coumaric acid. Similar results were obtained in BNC membranes containing EPP-AF^®^ microemulsion; the authors attributed this behavior to the polarity of the biomarkers [32]. Possibly due to its greater hydrophilicity, 50% of *p*-coumaric acid was released from all hydrogels in a period of 2 h to 3 h, while the same percentage of lipophilic artepillin C was released in an average time of 45 h among tested formulations.

In all hydrogels, artepillin C release was more gradual compared to that of *p*-coumaric acid. BNCH/P1 released both biomarkers faster than the other biomembranes, possibly due to the lower propolis concentration and, consequently, reduced interaction between the propolis compounds and BNC, favoring faster delivery. A higher content of biomarkers in more concentrated formulations can generate more chemical interactions between propolis molecules and the BNC matrix, such as hydrogen and Van der Waals bonds, trapping these compounds in the nanometric networks of BNC and hindering its release. This hypothesis corroborates MEV results, which show that the nanofibers are denser and more compacted in more concentrated formulations. The slower release rate, with an increase in propolis in the hydrogels, can be attributed to the greater number of intermolecular bonds [70].

The maximum release of *p*-coumaric acid reaches 90–100% in 21 h, in contrast to 75% for artepillin C in 72 h. Despite the difference between the biomarkers, both releases can be considered prolonged compared to other studies using BNC as a drug delivery system. Fontes et al., (2018) [71] produced BNC/carboxymethylcellulose composites and obtained a 70–80% delivery burst in 15 min, a significantly shorter time than that obtained in this work. The result of a longer release is interesting, considering the topical application of the formulations. Once the action of hydrogels is extended, it is possible to promote gradual bacterial inhibition, which can facilitate treatment and wound healing [72].

The release kinetics of propolis biomarkers from BNC hydrogels were determined based on mathematical functions that describe the release profile through correlation coefficients (r). The values obtained for each formulation are shown in Table 2.

According to the *r* values, it is possible to infer that both biomarkers follow the Higuchi model, which is characteristic of all BNC/propolis hydrogels. This profile suggests the diffusion type of biomarker release from the BNC matrix [73], which occurs mainly due to its insolubility [74].

#### 3.1.6. Mutagenic Potential

Table 3 shows the mean (M) and standard deviation (SD) of the number of revertants per plate and mutagenicity index (MI, values in parentheses) in TA 98, TA 100, TA97a, and TA 102 strains of *S. typhimurium* after treatment with hydrogels according to experiments without and with metabolic activation (−S9 and +S9, respectively).

All experiments presented MI values below 2.0, which indicates that the hydrogels did not induce a statistically significant increase in the number of revertants compared to the negative control (DMSO), both in the presence and absence of metabolic activation. BNCH/EPP3 generated more sensitivity to the used strains, as evidenced by the reduction in the number of revertants compared to the negative control and the decrease in MI values. Nevertheless, data demonstrate the absence of mutagenic activity of all formulations under the applied conditions. The non-mutagenic effect of propolis was confirmed in other works [53,75]. The positive control of each cell line produced the expected mutagenic response and was used to validate the susceptibility of the system to standard mutagens.

### 3.2. Characterization of Bacterial Nanocellulose Hydrogels Containing Propolis/Methylene Blue

#### 3.2.1. Spectroscopic Analysis

**Figure 5:**
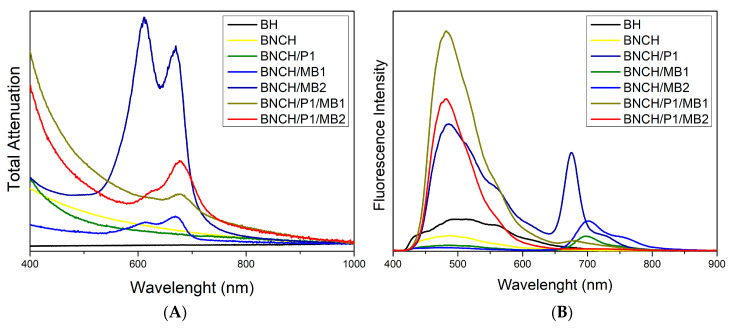
(**A**) UV-Vis and (**B**) fluorescence emission spectra of the hydrogels.

Figure 5 presents the UV-Vis and fluorescence emission spectra for the formulations prepared for analysis of photodynamic inactivation. The spectra of the BH and BNCH base formulations (Figure 5A) show no absorption band, which is expected due to the high transparency of the Natrosol^®^ hydrogel (BH) and the light scattering caused by the high content of cellulose nanofibers in the BNCH [76].

**Figure 6:**
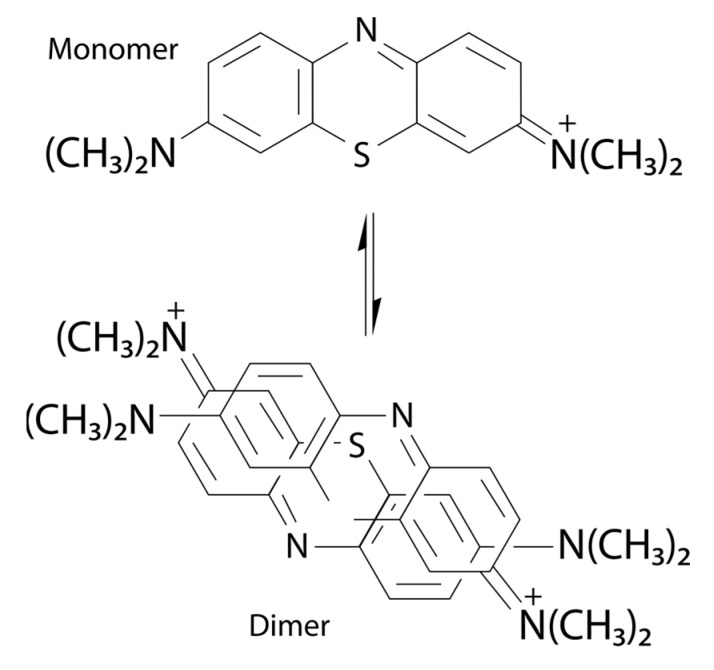
Chemical structure of methylene blue and its possible dimer.

BNC hydrogels containing MB present two main absorption bands, with maxima centered around 670 nm and 610 nm, with the highest and the lowest intensities, respectively, in the less concentrated formulation (BNCH/MB1). However, in the most concentrated formulation (BNCH/MB2), these bands have their intensities inverted, suggesting an MB dimerization process (Figure 6). According to the literature, MB is in balance between its monomeric and dimeric forms, which have maximum absorbance around 660 nm and 610 nm, respectively, similar to our results. At higher concentrations of MB, there is a tendency for dye aggregation, generating an equilibrium shift for the formation of dimers and band inversion [50]. In formulations containing MB and EPP-AF^®^, it is possible to observe a slight shift of the bands from 670 and 610 nm to 680 and 620 nm, respectively, probably caused by the interaction between MB and propolis.

The BNC/propolis hydrogel also does not present absorption bands in this region of the spectrum. Some previous studies on the ethanolic extract of propolis have reported maximum absorbance around 295 nm, as well as absorption of phenolic compounds under UV light [70,75]. However, this wavelength was not addressed in this work since the PDI application uses visible light, in this case at 660 nm due to the MB maximum absorbance.

In the fluorescence spectra of the formulations (Figure 5B), there are two main emission bands: one between 450 nm and 550 nm and another between 650 nm and 750 nm, corresponding to blue/green and red regions, respectively. Except for BNCH/MB1 and BNCH/MB2, all samples present an emission band around 500 nm. Only hydrogels containing MB have a band at 700 nm, suggesting the emission of the dye, which, in its pure state, emits fluorescence at 680 nm [77,78].

The band at 675 nm appears only in the spectrum of the BNCH/P1 formulation, which is assigned to emission from the propolis extract. In hydrogels containing EPP-AF^®^ and MB, the band at 675 nm appears more discrete, and bands around 700 nm are absent, which indicates a probable suppression of fluorescence due to the interaction of propolis with MB.

#### 3.2.2. Confocal Fluorescence Microscopy

**Figure 7:**
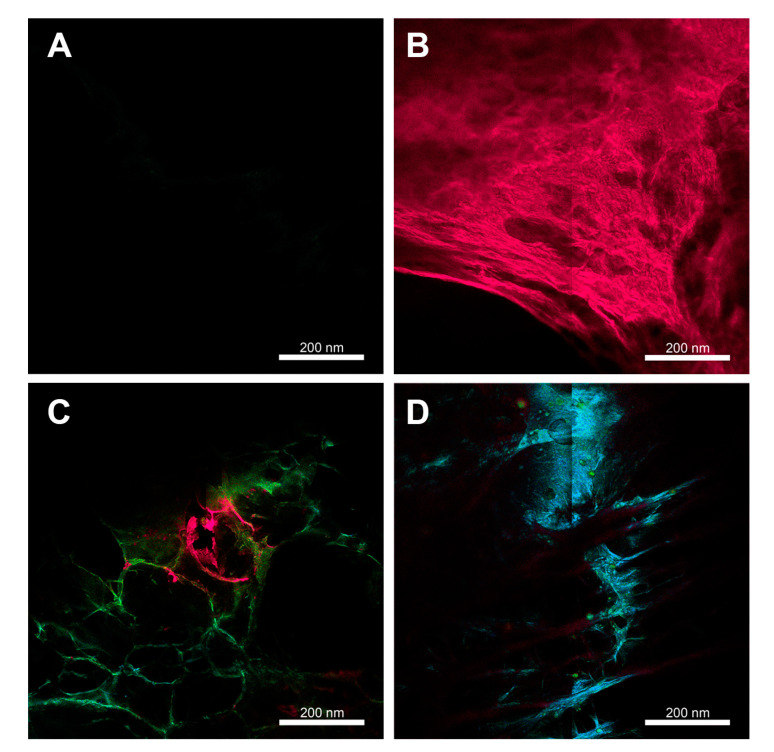
Confocal fluorescence microscopic images of the formulations: (**A**) BNCH, (**B**) BNCH/MB1, (**C**) BNCH/P1, and (**D**) BNCH/P1/MB1.

Images obtained by confocal fluorescence microscopy (Figure 7) are in accordance with the emission spectra described in the previous section. The formulations containing MB show fluorescence emission in red (Figure 7B), corresponding to the bands around 700 nm observed in the spectra. The BNCH/P1 image (Figure 7C) exposes the emission in the blue/green region, as well as the presence of some emission traces in the red region, which correspond to the bands around 500 and 675 nm, respectively. In the image of the formulation containing EPP-AF^®^ and MB (Figure 7D), the emission in the blue region is predominant, which corroborates the high emission observed in the spectra of the BNCH/P1/MB1 and BNCH/P1/MB2 hydrogels around 500 nm.

The images also corroborate the macroscopic aspect and the SEM images of the hydrogels, which have porous spaces in their structures. Porous materials contribute to the realization of PDT/PDI due to the need for the presence of oxygen for photodynamic reactions to occur and because they favor the incorporation of assets in their matrices [79].

### 3.3. Antimicrobial and Photodynamic Inactivation Assays

**Figure 8:**
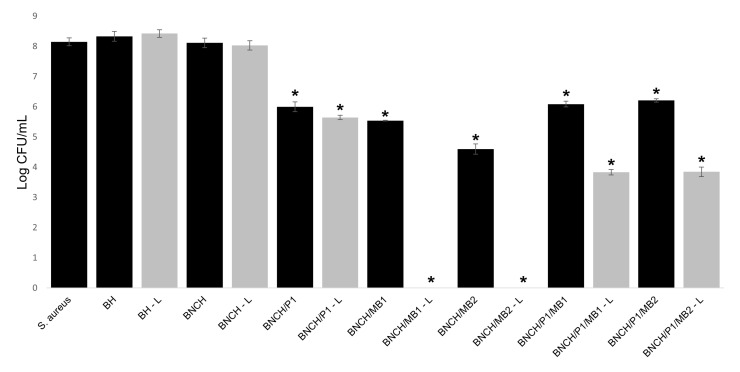
Antimicrobial activity of the formulations against *S. aureus* in protocols without light and after light exposure with a fluency of 50 J cm^−2^ at 660 nm (L). Results are expressed as mean inhibition ± SD. Statistical analysis was performed with one-way ANOVA, and post hoc Tukey’s test revealed a significant reduction in S. aureus (* *p* < 0.05 vs. control *S. aureus*).

Figure 8 and Table 4 show the results of the PDI treatment against *S. aureus* mediated by the MB present in BNC/propolis formulations. The BNC hydrogels containing propolis and/or MB were tested to compare antimicrobial photodynamic activity of isolated MB and MB in the presence of EPP-AF^®^, both in hydrogel form.

BNCH/MB1 and BNCH/MB2 formulations showed a significant reduction in *S. aureus* growth (2.61 ± 0.01 and 3.55 ± 0.17 log CFU/mL, respectively), even in the experiments without light (*p* < 0.05). This result confirms the toxicity of MB in the dark [80]. However, in the absence of light, the antimicrobial activity of BNCH/MB2 was significantly higher than that of BNCH/MB1, demonstrating the dose-dependent action of MB. In the presence of light, both hydrogels containing MB completely eradicated the microorganism (*p* = 0), suggesting that these are the most suitable formulations for PDI against *S. aureus*.

The microdilution results are in agreement with the disk diffusion experiment results (Table 4). It was possible to observe that BNC hydrogels containing only MB presented larger bacterial growth inhibition zones. The inhibition zone was especially significant in the most concentrated formulation (BNCH/MB2), the halo of which reached 5.0 ± 0.1 mm in the experiment with light irradiation, confirming the photodynamic effect of the formulations containing MB against *S. aureus*.

The photodynamic activity of MB occurs through type I reactions, which produce reactive oxygen species such as hydroxyl, and through type II reactions, which generate singlet oxygen. This species is considered the main cause of antimicrobial photodynamic activity, and the phenothiazine dye is well known for its high quantum yield of singlet oxygen [80,81]. Accordingly, MB-mediated PDI efficacy is well-founded and has been demonstrated in several studies and clinical applications, indicating a significant effect against a broad range of microorganisms, including resistant strains of *S. aureus* and *P. aeruginosa* [5].

Regarding the BNCH- and BH-based hydrogels, the formulation containing only propolis (BNCH/P1) significantly reduced bacterial growth (reduction of 2.15 log CFU/mL and inhibition zone of 3 ± 0.1 mm), but there was no significant difference between these formulations in the treatments with and without light (*p* > 0.05). This confirms that the antimicrobial activity of EPP-AF^®^ is not influenced by the presence of light [82,83]. In a similar work, BNC membranes containing 1.2% propolis, as well as the formulation tested in this experiment, were able to inhibit *S. aureus* growth, generating an inhibition zone of 8 ± 0.58 mm [20]. The antibacterial activity of propolis is mainly related to the action of flavonoids and cinnamic acid derivatives present in its composition. Different bacterial inhibition values are due to differences in compounds and their contents in different extracts [83].

Hydrogels containing MB associated with propolis (BNCH/P1/MB1 and BNCH/P1/MB2) had significantly reduced PDI efficacy (*p* < 0.05) compared to those containing only MB, maintaining values close to 2 log CFU/mL of reduction for both in the dark. After light irradiation, the reduction in microbial growth increased to 4.30 log CFU/mL. Despite the favorable results of these formulations for PDI application, they suggest that propolis decreased the photodynamic action of MB against the bacteria. This may be related to the shift in MB maximum absorbance in the presence of propolis, as demonstrated by the UV-Vis spectra, which may have reduced the formation of reactive species responsible for the inactivation of the microorganism. Unlike the results obtained in this work, Wang et al. (2017) [84] achieved an increased phototoxicity of PS protoporphyrin IX (PpIX) by combining it with propolis. The synergistic effect between them significantly decreased tumor volume and mass in a xenographic model compared to PS alone.

In addition to the absorbance shift, there is the possibility of a reaction between MB, which has a positive charge [51], and the hydroxyls of the phenolic compounds of propolis, which have a negative charge [85], causing the inactivation of some active substances. However, it is possible that propolis reacted with ^1^O_2_, acting as a sequestering agent and protecting bacteria from photodynamic effects.

The most accepted idea by the authors of this work regarding the reduction in the antimicrobial activity of BNCH/P/MB in relation to BNCH/MB concerns the turbidity of EPP-AF^®^ at the concentrations used. This may have hampered light transmission through formulations containing propolis, decreasing the amount of energy supplied to the PS, thereby reducing the formation of reactive species. The difficulty in performing antimicrobial tests for propolis has been reported in other works due to its turbidity [24]. However, to confirm this hypothesis, further tests with different concentrations of propolis are needed.

Nevertheless, all formulations containing EPP-AF^®^ and/or MB showed antimicrobial activity against *S. aureus* and may be possible future alternatives for wound care, since they may prevent or heal infections. The eradication of the microorganism by hydrogels containing MB after exposure to light expands the application of BNC formulations to PDI, supporting wound healing by eliminating infections.

## 4. Conclusions

New formulations of BNC hydrogels containing EPP-AF^®^ and MB photosensitizer were developed. The formulations maintained the BNC structure of a three-dimensional network of nanofibers, as well as its molecular organization and thermal stability. Furthermore, they presented pseudoplastic thixotropic behavior, making them suitable for topical applications. The quantity of propolis biomarkers in the formulations correspond to the concentration of the EPP-AF^®^ extract used, and their release was sustained for at least 20 h, representing a possible extended-release system. The obtained hydrogels did not present mutagenicity. In order to apply the formulations to photodynamic inactivation, BNC/propolis hydrogels containing MB were produced. Optical characterization showed maxima centered at 670 nm and 610 nm for pure MB formulations. In the most concentrated formulation, these bands have their intensities inverted, suggesting an MB dimerization process. There was a slight shift in the maximum absorbance of the dye with propolis to 680 nm and 620 nm, which may have led to a decrease in the PDI efficacy. Nevertheless, all formulations containing propolis and/or MB were able to reduce S. aureus growth on some scale. As an advantage, the BNC/MB hydrogels completely inhibited the growth of the bacteria after exposure to light, demonstrating great efficacy for application in PDI. We conclude that the new formulation of BNC produced in this study can potentially support wound healing by treating or preventing infections.

## Figures and Tables

**Table 1:** Chemical composition of EPP-AF^®^ biomarkers in propolis hydrogels (average ± SD).

Biomarker	Mean Concentration (mg/g) ± SD		
	BNCH/P1	BNCH/P2	BNCH/P3
*p*-Coumaric acid	0.151 ± 0.0008	0.336 ± 0.0160	0.453 ± 0.0042
Artepillin C	0.782 ± 0.0154	1.583 ± 0.0740	2.171 ± 0.0404
Baccharin	0.103 ± 0.00262	0.17 ± 0.00738	0.211 ± 0.0034

**Table 2:** Correlation coefficients (*r*) of the linear fraction of the biomarker release curves.

Biomarker	Formulation	Correlation Coefficients (*r*)		
		Zero-Order µg/h	Higuchi Model µg/√h	First-Order Log(µg)/h
*p*-Coumaric acid	BNCH/P1	0.566	0.720	0.554
	BNCH/P2	0.717	0.839	0.684
	BNCH/P3	0.472	0.617	0.418
Artepillin C	BNCH/P1	0.853	0.945	0.771
	BNCH/P2	0.969	0.995	0.872
	BNCH/P3	0.917	0.980	0.750

**Table 3:** Number of revertants/plate and MI from BNC formulations containing EPP-AF^®^. Positive control (C+): (a) 4-nitro-o-phenylenediamine (10.0 μg/plate–TA98, TA97a); (b) sodium azide (1.25 μg/plate–TA100); (c) mitomycin C (0.5 μg/placa–TA102) in the absence of S9 and (d) 2-aminoanthracene (1.25 μg/plate–TA98, TA97a e TA100); (e) 2-aminofluorene (10 μg/ placa–TA102) in the presence of S9. Negative control: dimethyl sulfoxide (DMSO–100 μL/ plate).

Treatment	Number of Revertants (M ± SD)/Plate and MI							
	TA98		TA100		TA102		TA97a	
µg /Plate	−S9	+S9	−S9	+S9	−S9	+S9	−S9	+S9
DMSO	16 ± 0	26 ± 3	102 ± 8	93 ± 11	228 ± 22	289 ± 31	146 ± 10	120 ± 18
BNCH	14 ± 1 (0.88)	27 ± 2 (1.02)	95 ± 5 (0.93)	115 ± 14 (1.23)	231 ± 12 (1.01)	254 ± 25 (0.88)	133 ± 17 (0.91)	129 ± 11 (1.08)
BNCH/P3	10 ± 2 (0.59)	16 ± 2 (0.62)	78 ± 3 (0.77)	90 ± 18 (0.97)	128 ± 7 (0.56)	250 ± 33 (0.87)	88 ± 2 (0.60)	102 ± 17 (0.85)
BNCH/P2	15 ± 1 (0.94)	21 ± 1 (0.81)	90 ± 4 (0.89)	95 ± 8 (1.02)	204 ± 28 (0.90)	270 ± 20 (0.94)	116 ± 14 (0.80)	113 ± 11 (0.95)
BNCH/P1	18 ± 1 (1.13)	27 ± 2 (1.02)	98 ± 9 (0.96)	98 ± 4 (1.05)	257 ± 35 (1.13)	308 ± 35 (1.07)	175 ± 22 (1.20)	134 ± 13 (1.12)
C +	599 ± 56 (a),*	927 ± 89 (d),*	1266 ± 152 (b),*	1886 ± 175 (d),*	1882 ± 103 (c)*	1266 ± 101 (e),*	612 ± 62 (a),*	1756 ± 135 (d),*

* Statistically different from the DMSO (*p* < 0.05) according to one-way ANOVA test followed by the Dunnett’s post hoc test. The assay was performed in triplicate.

**Table 4:** Mean inhibition zone ± SD of *S. aureus* growth after treatment with the formulations in experiments in the dark and with light irradiation (50 J cm^−2^ of red light at 660 nm). Statistical analysis was performed with one-way ANOVA, post hoc Tukey’s test revealed a significant increase in the inhibition zone (* *p* < 0.05 vs. control *S. aureus*).

Mean Inhibition Halo (mm) ± SD		
Samples	Dark	Light (660 nm–50 J cm^−2^)
*S. aureus*	0.0 ± 0.0	0.0 ± 0.0
BH	1.0 ± 0.1	0.0 ± 0.0
BNCH	0.0 ± 0.0	0.0 ± 0.0
BNCH/P1 *	3.0 ± 0.0	3.0 ± 0.0
BNCH/MB1 *	3.0 ± 0.1	4.0 ± 0.1
BNCH/MB2 *	5.0 ± 0.1	5.0 ± 0.1
BNCH/P1/MB1 *	2.0 ± 0.0	3.0 ± 0.1
BNCH/P1/MB2 *	2.0 ± 0.1	2.0 ± 0.1

## Data Availability

The data presented in this study are available on request from the corresponding authors.

## References

[1] Broughton G., Janis J.E., Attinger C.E. (2006). The Basic Science of Wound Healing. Plast. Reconstr. Surg..

[2] Broughton G., Janis J.E., Attinger C.E. (2006). Wound Healing: An Overview. Plast. Reconstr. Surg..

[3] Guo S., DiPietro L.A. (2010). Critical Review in Oral Biology & Medicine: Factors Affecting Wound Healing. J. Dent. Res..

[4] Healy B., Freedman A. (2006). ABC of Wound Healing:Infections. Bmj.

[5] Shen X., Dong L., He X., Zhao C., Zhang W., Li X., Lu Y. (2020). Treatment of Infected Wounds with Methylene Blue Photodynamic Therapy: An Effective and Safe Treatment Method. Photodiagnosis Photodyn. Ther..

[6] Coelho V.H.M., Alvares L.D., Carbinatto F.M., de Aquino Junior A.E., Ramirez Angarita D.P., Bagnato V.S. (2017). Photodynamic Therapy, Laser Therapy and Cellulose Membrane for the Healing of Venous Ulcers: Results of a Pilot Study. J. Nurs. Care.

[7] Boateng J., Catanzano O. (2015). Advanced Therapeutic Dressings for Effective Wound Healing—A Review. J. Pharm. Sci..

[8] Han G., Ceilley R. (2017). Chronic Wound Healing: A Review of Current Management and Treatments. Adv. Ther..

[9] Pereira G.B., Ferreira V., Martins G.L., Oliveira Barud H.G., Balestra F.L., Banhos E., Parro M.C., Jacon J.C., Barud H.S., Aquino Junior A.E., Carbinatto F.M., Coelho V.H.M., Bagnato V.S. (2019). Tratamentos Tópicos Com Ênfase Em Principais Curativos-Cap. 23. Feridas, Um Desafio Para a Saúde Pública.

[10] Gürgen M. (2014). Excess Use of Antibiotics in Patients with Non-Healing Ulcers. Ewma J..

[11] Czaja W., Krystynowicz A., Bielecki S., Brown R.M. (2006). Microbial Cellulose—The Natural Power to Heal Wounds. Biomaterials.

[12] Czaja W.K., Young D.J., Kawecki M., Brown R.M. (2007). The Future Prospects of Microbial Cellulose in Biomedical Applications. Biomacromolecules.

[13] Loh E.Y.X., Mohamad N., Fauzi M.B., Ng M.H., Ng S.F., Mohd Amin M.C.I. (2018). Development of a Bacterial Cellulose-Based Hydrogel Cell Carrier Containing Keratinocytes and Fibroblasts for Full-Thickness Wound Healing. Sci. Rep..

[14] Klemm D., Heublein B., Fink H.P., Bohn A. (2005). Cellulose: Fascinating Biopolymer and Sustainable Raw Material. Angew. Chem. Int. Ed..

[15] Klemm D., Cranston E.D., Fischer D., Gama M., Kedzior S.A., Kralisch D., Kramer F., Kondo T., Lindström T., Nietzsche S. (2018). Nanocellulose as a Natural Source for Groundbreaking Applications in Materials Science: Today’s State. Mater. Today.

[16] Chang W.S., Chen H.H. (2016). Physical Properties of Bacterial Cellulose Composites for Wound Dressings. Food Hydrocoll..

[17] Barud H.S., Regiani T., Marques R.F.C., Lustri W.R., Messaddeq Y., Ribeiro S.J.L. (2011). Antimicrobial Bacterial Cellulose-Silver Nanoparticles Composite Membranes. J. Nanomater..

[18] Horue M., Cacicedo M.L., Fernandez M.A., Rodenak-Kladniew B., Torres Sánchez R.M., Castro G.R. (2020). Antimicrobial Activities of Bacterial Cellulose—Silver Montmorillonite Nanocomposites for Wound Healing. Mater. Sci. Eng. C.

[19] Popa L., Truşcă R.D., Ilie C.-I., Tipela R.E., Ficai D., Oprea O., Stoica-Guzun A., Ficai A., Ditu L.-M. (2020). Antibacterial Activity of Bacterial Cellulose Loaded. Molecules.

[20] Barud H.D.S., de Araújo Júnior A.M., Saska S., Mestieri L.B., Campos J.A.D.B., de Freitas R.M., Ferreira N.U., Nascimento A.P., Miguel F.G., Vaz M.M.D.O.L.L. (2013). Antimicrobial Brazilian Propolis (EPP-AF) Containing Biocellulose Membranes as Promising Biomaterial for Skin Wound Healing. Evid. Based Complement. Altern. Med..

[21] Kabir S.M.F., Sikdar P.P., Haque B., Bhuiyan M.A.R., Ali A., Islam M.N. (2018). Cellulose-Based Hydrogel Materials: Chemistry, Properties and Their Prospective Applications. Prog. Biomater..

[22] Park Y.K., Alencar S.M., Aguiar C.L. (2002). Botanical Origin and Chemical Composition of Brazilian Propolis. Agric. Food Chemisry.

[23] Cauich-Kumul R., Segura Campos M.R. (2019). Bee Propolis: Properties, Chemical Composition, Applications and Potencial Health Effects. Bioactive Compounds.

[24] Berretta A.A., Nascimento A.P., Pires Bueno P.C., Lima Leite Vaz MM D.O., Marchetti J.M. (2012). Propolis Standardized Extract (EPP-AF^®^), an Innovative Chemically and Biologically Reproducible Pharmaceutical Compound for Treating Wounds. Int. J. Biol. Sci..

[25] Santos P.B. (2020). do R.E. dos; Ávila, D. da S.; Ramos, L. de P.; Yu, A.R.; Santos, C.E. da R.; Berretta, A.A.; Camargo, S.E.A.; Oliveira, J.R. de; Oliveira, L.D. de Effects of Brazilian Green Propolis Extract on Planktonic Cells and Biofilms of Multidrug-Resistant Strains of Klebsiella Pneumoniae and Pseudomonas Aeruginosa. Biofouling.

[26] Paulino N., Abreu S.R.L., Uto Y., Koyama D., Nagasawa H., Hori H., Dirsch V.M., Vollmar A.M., Scremin A., Bretz W.A. (2008). Anti-Inflammatory Effects of a Bioavailable Compound, Artepillin C, in Brazilian Propolis. Eur. J. Pharmacol..

[27] Almeida-Junior S., Pereira D.V., Ferreira T.M.F., Freitas R.A., Silva C.C., Santos M.F.C., Borges C.H.G., Andrade e Silva M., Ambrósio S.R., Bastos J.K. (2020). Anti-Inflammatory and Antinociceptive Effects of Kaempferide from the Brazilian Green Propolis. Res. Soc. Dev..

[28] Diniz D.P., Lorencini D.A., Berretta A.A., Cintra M.A.C.T., Lia E.N., Jordão A.A., Coelho E.B. (2020). Antioxidant Effect of Standardized Extract of Propolis (EPP-AF^®^) in Healthy Volunteers: A “before and After” Clinical Study. Evid. Based Complement. Altern. Med..

[29] Bhargava P., Grover A., Nigam N., Kaul A., Doi M., Ishida Y., Kakuta H., Kaul S.C., Terao K., Wadhwa R. (2018). Anticancer Activity of the Supercritical Extract of Brazilian Green Propolis and Its Active Component, Artepillin C: Bioinformatics and Experimental Analyses of Its Mechanisms of Action. Int. J. Oncol..

[30] Quintino R.L., Reis A.C., Fernandes C.C., Martins C.H.G., Colli A.C., Crotti A.E.M., Squarisi I.S., Ribeiro A.B., Tavares D.C., Miranda M.L.D. (2020). Brazilian Green Propolis: Chemical Composition of Essential Oil and Their In Vitro Antioxidant, Antibacterial and Antiproliferative Activities. Braz. Arch. Biol. Technol..

[31] Ebadi P., Fazeli M. (2021). Evaluation of the Potential in Vitro Effects of Propolis and Honey on Wound Healing in Human Dermal Fibroblast Cells. South Afr. J. Bot..

[32] Marquele-Oliveira F., da Silva Barud H., Torres E.C., Machado R.T.A., Caetano G.F., Leite M.N., Frade M.A.C., Ribeiro S.J.L., Berretta A.A. (2019). Development, Characterization and Pre-Clinical Trials of an Innovative Wound Healing Dressing Based on Propolis (EPP-AF^®^)-Containing Self-Microemulsifying Formulation Incorporated in Biocellulose Membranes. Int. J. Biol. Macromol..

[33] Hawkins D., Abrahamse H. (2006). Effect of Multiple Exposures of Low-Level Laser Therapy on the Cellular Responses of Wounded Human Skin Fibroblasts. Photomed. Laser Surg..

[34] Rossi F., Magni G., Tatini F., Banchelli M., Cherchi F., Rossi M., Coppi E., Pugliese A.M., Rossi Degl’Innocenti D., Alfieri D. (2021). Photobiomodulation of Human Fibroblasts and Keratinocytes with Blue Light: Implications in Wound Healing. Biomedicines.

[35] Khan I., Rahman S.U., Tang E., Engel K., Hall B., Kulkarni A.B., Arany P.R. (2021). Accelerated Burn Wound Healing with Photobiomodulation Therapy Involves Activation of Endogenous Latent TGF-Β1. Sci. Rep..

[36] Mosca R.C., Ong A.A., Albasha O., Bass K., Arany P. (2019). Photobiomodulation Therapy for Wound Care: A Potent, Noninvasive, Photoceutical Approach. Adv. Ski. Wound Care.

[37] Grecco C., Moriyama L.T. (2015). Princípios Básicos Em Terapia Fotodinâmica. Terapia Fotodinâmica Dermatológica: Programa TFD Brasil.

[38] Castano A.P., Demidova T.N., Hamblin M.R. (2004). Mechanisms in Photodynamic Therapy: Part One—Photosensitizers, Photochemistry and Cellular Localization. Photodiagnosis Photodyn. Ther..

[39] Kwiatkowski S., Knap B., Przystupski D., Saczko J., Kędzierska E., Knap-Czop K., Kotlińska J., Michel O., Kotowski K., Kulbacka J. (2018). Photodynamic Therapy—Mechanisms, Photosensitizers and Combinations. Biomed. Pharmacother..

[40] Dolmans D.E., Fukumura D., Jain R.K. (2003). Photodynamic Therapy for Cancer. Nat. Rev. Cancer.

[41] Yano T., Wang K.K. (2020). Photodynamic Therapy for Gastrointestinal Cancer. Photochem. Photobiol..

[42] Huang L., Dai T., Hamblin M.R., Gomer C. (2010). Antimicrobial Photodynamic Inactivation and Photodynamic Therapy for Infections. Photodynamic Therapy: Methods in Molecular Biology.

[43] Hamblin M.R. (2016). Antimicrobial Photodynamic Inactivation: A Bright New Technique to Kill Resistant Microbes. Curr. Opin. Microbiol..

[44] Blanco K.C., Inada N.M., Carbinatto F.M., Giusti A.L., Bagnato V.S. (2017). Treatment of Recurrent Pharyngotonsillitis by Photodynamic Therapy. Photodiagnosis Photodyn. Ther..

[45] Carmello J.C., Alves F., Basso F.G., de Souza Costa C.A., Bagnato V.S., Mima E.G.D.O., Pavarina A.C. (2016). Treatment of Oral Candidiasis Using Photodithazine^®^-Mediated Photodynamic Therapy in Vivo. PLoS ONE.

[46] Da Silva A.P., Kurachi C., Bagnato V.S., Inada N.M. (2013). Fast Elimination of Onychomycosis by Hematoporphyrin Derivative-Photodynamic Therapy. Photodiagnosis Photodyn. Ther..

[47] Kharkwal G.B., Sharma S.K., Huang Y.Y., Dai T., Hamblin M.R. (2011). Photodynamic Therapy for Infections: Clinical Applications. Lasers Surg. Med..

[48] Nezhadi J., Eslami H., Fakhrzadeh V., Moaddab S.R., Zeinalzadeh E., Kafil H.S. (2019). Photodynamic Therapy of Infection in Burn Patients. Rev. Med. Microbiol..

[49] Paolillo F.R., Rodrigues P.G.S., Bagnato V.S., Alves F., Pires L., Corazza A.V. (2021). The Effect of Combined Curcumin-Mediated Photodynamic Therapy and Artificial Skin on Staphylococcus Aureus–Infected Wounds in Rats. Lasers Med. Sci..

[50] Tardivo J.P., del Giglio A., de Oliveira C.S., Gabrielli D.S., Junqueira H.C., Tada D.B., Severino D., de Fátima Turchiello R., Baptista M.S. (2005). Methylene Blue in Photodynamic Therapy: From Basic Mechanisms to Clinical Applications. Photodiagnosis Photodyn. Ther..

[51] Calixto G.M.F., Bernegossi J., de Freitas L.M., Fontana C.R., Chorilli M., Grumezescu A.M. (2016). Nanotechnology-Based Drug Delivery Systems for Photodynamic Therapy of Cancer: A Review. Molecules.

[52] Maron D.M., Ames B.N. (1983). Revised Methods for the Salmonella Mutagenicity Test. Mutat. Res. Environ. Mutagen. Relat. Subj..

[53] Resende F.A., Munari C.C., de Azevedo Bentes Monteiro Neto M., Tavares D.C., Bastos J.K., da Silva Filho A.A., Varanda E.A. (2012). Comparative Studies of the (Anti) Mutagenicity of Baccharis Dracunculifolia and Artepillin C by the Bacterial Reverse Mutation Test. Molecules.

[54] Mortelmans K., Zeiger E. (2000). The Ames Salmonella/Microsome Mutagenicity Assay. Acarologia.

[55] de Paula Campos C., de Paula D’Almeida C., Nogueira M.S., Moriyama L.T., Pratavieira S., Kurachi C. (2017). Fluorescence Spectroscopy in the Visible Range for the Assessment of UVB Radiation Effects in Hairless Mice Skin. Photodiagnosis Photodyn. Ther..

[56] Romano R.A., Pratavieira S., da Silva A.P., Kurachi C., Guimarães F.E.G. Multispectral Confocal Microscopy Images and Artificial Neural Nets to Monitor the Photosensitizer Uptake and Degradation in Candida Albicans Cells. Proceedings of the European Conference on Biomedical Optics.

[57] de Oliveira Barud H.G., da Silva R.R., da Silva Barud H., Tercjak A., Gutierrez J., Lustri W.R., de Oliveira O.B., Ribeiro S.J.L. (2016). A Multipurpose Natural and Renewable Polymer in Medical Applications: Bacterial Cellulose. Carbohydr. Polym..

[58] Pacheco G., da Silva Filho E.C., Machado R.T.A., Ribeiro S.J.L., Meneguin A.B., Barud H.D.S., Nogueira C.R., Silva M.C.C., Trovatti E. (2017). Development and Characterization of Bacterial Cellulose Produced by Cashew Tree Residues as Alternative Carbon Source. Ind. Crops Prod..

[59] Radovanović V., Vlainić J., Hanžić N., Ukić P., Oršolić N., Baranović G., Jembrek M.J. (2019). Neurotoxic Effect of Ethanolic Extract of Propolis in the Presence of Copper Ions Is Mediated through Enhanced Production of ROS and Stimulation of Caspase-3/7 Activity. Toxins.

[60] Oliveira R.N., McGuinness G.B., Rouze R., Quilty B., Cahill P., Soares G.D.A., Thiré R.M.S.M. (2015). PVA Hydrogels Loaded with a Brazilian Propolis for Burn Wound Healing Applications. J. Appl. Polym. Sci..

[61] Mohammadkazemi F., Azin M., Ashori A. (2015). Production of Bacterial Cellulose Using Different Carbon Sources and Culture Media. Carbohydr. Polym..

[62] Gong T., Hou Y., Yang X., Guo Y. (2019). Gelation of Hydroxyethyl Cellulose Aqueous Solution Induced by Addition of Colloidal Silica Nanoparticles. Int. J. Biol. Macromol..

[63] Rocha B.A., Rodrigues M.R., Bueno P.C.P., de Mello Costa-Machado A.R., de Oliveira Lima Leite Vaz M.M., Nascimento A.P., Barud H.S., Berretta-Silva A.A. (2012). Preparation and Thermal Characterization of Inclusion Complex of Brazilian Green Propolis and Hydroxypropyl-β-Cyclodextrin: Increased Water Solubility of the Chemical Constituents and Antioxidant Activity. J. Therm. Anal. Calorim..

[64] Barud H.S., Assunção R.M.N., Martines M.A.U., Dexpert-Ghys J., Marques R.F.C., Messaddeq Y., Ribeiro S.J.L. (2008). Bacterial Cellulose-Silica Organic-Inorganic Hybrids. J. Solgel Sci. Technol..

[65] Corrêa N.M., Camargo Júnior F.B., Ignácio R.F., Leonardi G.R. (2005). Avaliação Do Comportamento Reológico de Diferentes Géis Hidrofílicos. Rev. Bras. De Ciências Farm..

[66] Carbinatto F.M., Sábio R.M., Meneguin A.B., Cestari S.E., Cruz S.A., Barud H.S. (2018). Bacterial Cellulose-Based Hydrogel for Wound Healing: Characterization and in Vitro Evaluation. Int. J. Adv. Med. Biotechnol..

[67] Schramm L.L. (2006). Emulsions, Foams, and Suspensions: Fundamentals and Applications.

[68] Lee C.H., Moturi V., Lee Y. (2009). Thixotropic Property in Pharmaceutical Formulations. J. Control. Release.

[69] Carvalho F.C., Calixto G., Hatakeyama I.N., Luz G.M., Gremião M.P.D., Chorilli M. (2013). Rheological, Mechanical, and Bioadhesive Behavior of Hydrogels to Optimize Skin Delivery Systems. Drug Dev. Ind. Pharm..

[70] De Lima G.G., de Souza R.O., Bozzi A.D., Poplawska M.A., Devine D.M., Nugent M.J.D. (2016). Extraction Method Plays Critical Role in Antibacterial Activity of Propolis-Loaded Hydrogels. J. Pharm. Sci..

[71] Fontes M.D.L., Meneguin A.B., Tercjak A., Gutierrez J., Cury B.S.F., Cury F., dos Santos A.M., Ribeiro S.J.L. (2018). Effect of in Situ Modification of Bacterial Cellulose with Carboxymethylcellulose on Its Nano/Microstructure and Methotrexate Release Properties. Carbohydr. Polym..

[72] Kachel E., Moshkovitz Y., Sternik L., Sahar G., Grosman-Rimon L., Belotserkovsky O., Reichart M., Stark Y., Emanuel N. (2020). Local Prolonged Release of Antibiotic for Prevention of Sternal Wound Infections Postcardiac Surgery—A Novel Technology. J. Card. Surg..

[73] Parente M.E., Ochoa Andrade A., Ares G., Russo F., Jiménez-Kairuz A. (2015). Bioadhesive Hydrogels for Cosmetic Applications. Int. J. Cosmet. Sci..

[74] Lopes C.M., Manuel J., Lobo S., Costa P. (2005). Formas Farmacêuticas de Liberação Modificada: Polímeros Hidrifílicos. Rev. Bras. De Cienc. Farm. Braz. J. Pharm. Sci..

[75] Maldonado L., Marcinkevicius K., Borelli R., Gennari G., Salomón V., Isla M.I., Vera N., Borelli V. (2018). Differentiation of Argentine Propolis from Different Species of Bees and Geographical Origins by UV Spectroscopy and Chemometric Analysis. J. Saudi Soc. Agric. Sci..

[76] Legnani C., Barud H.S., Caiut J.M.A., Calil V.L., Maciel I.O., Quirino W.G., Ribeiro S.J.L., Cremona M. (2019). Transparent Bacterial Cellulose Nanocomposites Used as Substrate for Organic Light-Emitting Diodes. J. Mater. Sci. Mater. Electron..

[77] Li L., Liu J., Yang X., Peng Z., Liu W., Xu J., Tang J., He X. (2015). ChemComm. Chamical Commun..

[78] Selvam S., Sarkar I., Selvam S., Sarkar I. (2016). Author’s Accepted Manuscript Molecular Mechanics Based Approach Bile Salt Induced Solubilization of Methylene Blue: Study on Methylene Blue Fluorescence and Molecular Mechanics Based Approach. J. Pharm. Anal..

[79] Zhang X., Xia L., Chen X., Chen Z., Wu F. (2017). Hydrogel-Based Phototherapy for Fighting Cancer and Bacterial Infection. Sci. China Mater..

[80] Luengas S.L.P., Marin G.H., Aviles K., Acuña R.C., Roque G., Nieto F.R., Sanchez F., Tarditi A., Rivera L., Mansilla E. (2014). Enhanced Singlet Oxygen Production by Photodynamic Therapy and a Novel Method for Its Intracellular Measurement. Cancer Biother. Radiopharm..

[81] Vecchio D., Gupta A., Huang L., Landi G., Avci P., Rodas A., Hamblina M.R. (2015). Bacterial Photodynamic Inactivation Mediated by Methylene Blue and Red Light Is Enhanced by Synergistic Effect of Potassium Iodide. Antimicrob. Agents Chemother..

[82] Pei K., Ou J., Huang J., Ou S. (2016). P-Coumaric Acid and Its Conjugates: Dietary Sources, Pharmacokinetic Properties and Biological Activities. J. Sci. Food Agric..

[83] Khoshnevisan K., Maleki H., Samadian H., Doostan M., Reza M. (2019). Antibacterial and Antioxidant Assessment of Cellulose Acetate/Polycaprolactone Nano Fibrous Mats Impregnated with Propolis. Int. J. Biol. Macromol..

[84] Wang C.-C., Wang Y.-X., Yu N.-Q., Hu D., Wang X.-Y., Chen X.-G., Liao Y.-W., Yao J., Wang H., He L. (2017). Brazilian Green Propolis Extract Synergizes with Protoporphyrin IX-Mediated Photodynamic Therapy via Enhancement of Intracellular Accumulation of Protoporphyrin IX and Attenuation of NF-ΚB and COX-2. Molecules.

[85] Volpi N. (2004). Separation of Flavonoids and Phenolic Acids from Propolis by Capillary Zone Electrophoresis. Electrophoresis.

